# Emergency transanal total mesorectal excision for perforated rectal cancer: a two-case series

**DOI:** 10.1186/s40792-022-01480-z

**Published:** 2022-06-22

**Authors:** Hiroya Enomoto, Katsuhito Suwa, Nana Takeuchi, Yuhei Tsukazaki, Takuro Ushigome, Tomoyoshi Okamoto, Ken Eto

**Affiliations:** 1 Department of Surgery, The Jikei University Daisan Hospital, 4-11-1 Izumihoncho, Komae, Tokyo, 201-8601 Japan; 2 Department of Surgery, The Jikei University Hospital, 3-25-8 Nishishinbashi, Minato-ku, Tokyo, 105-8461 Japan

**Keywords:** Perforated colorectal cancer, TaTME, Rectal cancer

## Abstract

**Background:**

Surgery for perforated rectal cancer is technically difficult because of paralytic dilatation due to generalized fecal peritonitis, the presence of a bulky tumor, and fecal retention due to obstruction. Transanal total mesorectal excision (TaTME) is the latest minimally invasive transanal technique pioneered to facilitate difficult pelvic dissections. It can provide a good surgical field linearly from the perineal side and reduce manipulations from the intraabdominal side. Here, we present two cases of emergency TaTME performed for perforated rectal cancer.

**Case presentation:**

The patients were a 38-year-old female and a 75-year-old male. They were diagnosed with perforated rectal cancer and were in a state of septic shock. Emergency Hartmann’s procedure was performed in both cases. Intraoperative findings showed fecal contamination of the entire abdomen and dilated intestines and bulky tumors with perforation. The female patient had multiple uterine fibroids, and the male patient had an enlarged prostate. For both patients, dissection of the mesorectum to the anal side of the tumor and transection of the rectum on the anal side of the tumor via a linear stapler were considered difficult because of the insufficient surgical field of view into the pelvis. Therefore, a two-team approach with TaTME was adopted. En bloc resection of the rectum was completed by collaboration of the abdominal team and the transanal team, and the autonomic nerves were successfully preserved. Finally, the specimens were resected, and the anal edge of the rectum was closed with a purse-string suture by the transanal team. Although these two cases were emergency surgeries in difficult situations, the cancer lesions were successfully and safely removed without involvement of the resection margin.

**Conclusions:**

This is the first report of emergency TaTME. Although these cases were emergency operations in a situation where it was difficult to pursue radical resection—and often times in these situations, the operation may end with only stoma creation—the specimens were safely resected. Emergency TaTME is a useful procedure for treatment of perforated rectal cancer.

## Background

Perforated colorectal cancer, which frequently leads to septic shock, is an extremely serious condition with high mortality and morbidity rates [1–3]. Patients undergo surgery with the dual life threats of a malignant disease and sepsis due to perforated peritonitis. Moreover, the operation is complicated by factors, such as paralytic bowel dilatation from generalized fecal peritonitis, bulkiness of the tumor, and fecal retention due to obstruction.

Total mesorectal excision (TME) for rectal cancer is a widely performed procedure. However, the transection of the rectum with a linear stapler becomes difficult because of the limitations either in visualizing or in handling the linear cutter in the deep pelvic space, especially for male patients with narrow pelvises, extreme obesity, bulky tumors, large uterine fibroids, enlarged prostates, or tumors in the lower rectum. Transanal total mesorectal excision (TaTME) is the latest minimally invasive transanal technique pioneered to facilitate difficult pelvic dissections [4]. Although it is difficult to pursue radical resection for perforated rectal cancer, and the operation may end with only stoma creation without tumor resection, the application of TaTME allows for a relatively short operation time while ensuring the ability to cure the cancer. At our institution, we have adopted and have been promoting the two-team approach with TaTME for difficult cases of perforated rectal carcinoma with poor surgical views while maintaining speed and safety, which are both important factors when performing emergency surgery. To the best of our knowledge, this is the first report of emergency TaTME.

## Case presentation

**Fig. 1:**
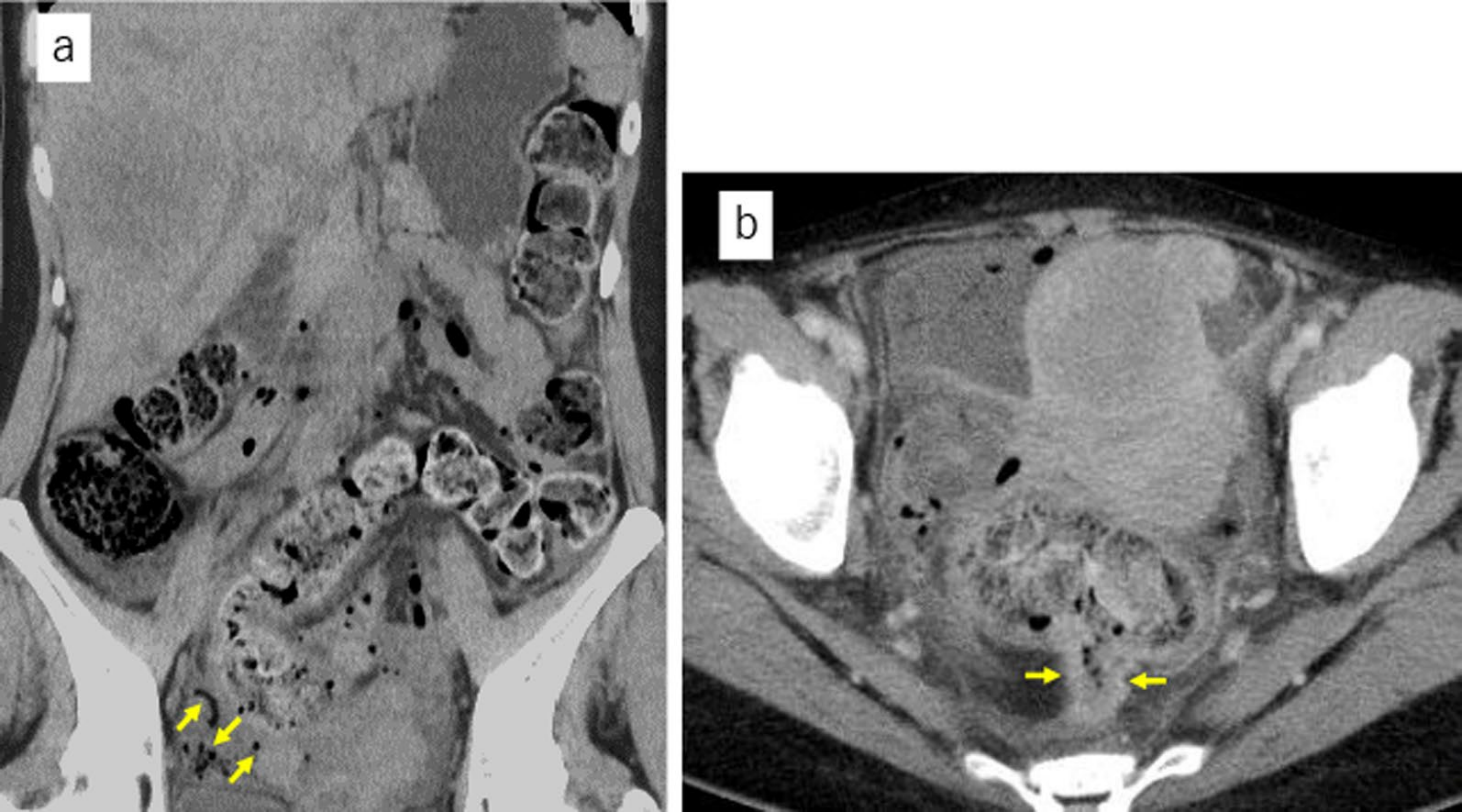
Computed tomography in Case 1. **a** Computed tomography showed a large amount of fecal mass in the colon and free air (yellow arrows) in the pelvis. A large mass was observed in the liver. **b** There was wall thickening in the rectum (yellow arrows), and the oral side of the rectum was dilated and had a fecal mass. Multiple uterine fibroids were seen, and ascites with free air was observed around them

*Case 1*: A 38-year-old female patient was brought to the hospital urgently with complaints of abdominal pain and high fever and was diagnosed with obstructive rectal cancer 8 cm from the anal verge, with multiple liver metastases as well as multiple uterine fibroids (Fig. 1). The cancer had perforated, causing panperitonitis, and she was in a state of septic shock. Emergency laparoscopic Hartmann’s procedure was performed. A 12-mm trocar was inserted through an umbilical incision, and then the pneumoperitoneum was created. Another 12-mm trocar was placed in the right lower quadrant, and three 5-mm trocars were inserted in the left lower quadrant and bilateral lateral regions.

Laparoscopic examination revealed fecal contamination of the entire abdomen, a dilated intestinal tract, and multiple uterine fibroids. A bulky tumor was observed at the peritoneal reflexion, and the perforation was on the anterior wall of the tumor. Resection of the perforated rectum along with the tumor was considered necessary. The uterus was lifted up to the abdominal wall; however, the surgical view in the pelvis was poor, and it was judged that the dissection of the mesorectum to the anal side of the tumor and the transection of the rectum on the anal side of the tumor with a linear stapler would be technically difficult.

**Fig. 2:**
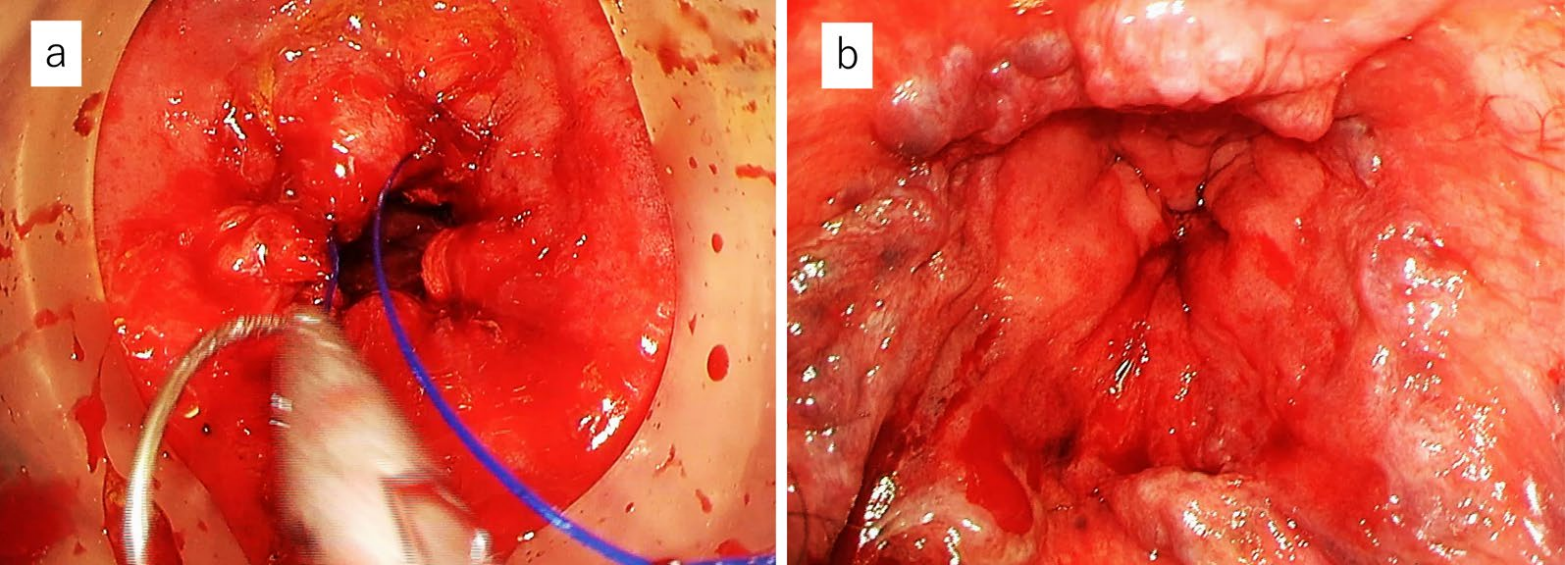
Distal purse-string suture from below. **a** After removal of the specimen, the anal edge of the rectum was closed with a purse-string suture by a transanal team. **b** A photograph taken after completion of the distal purse-string suture

**Fig. 3:**
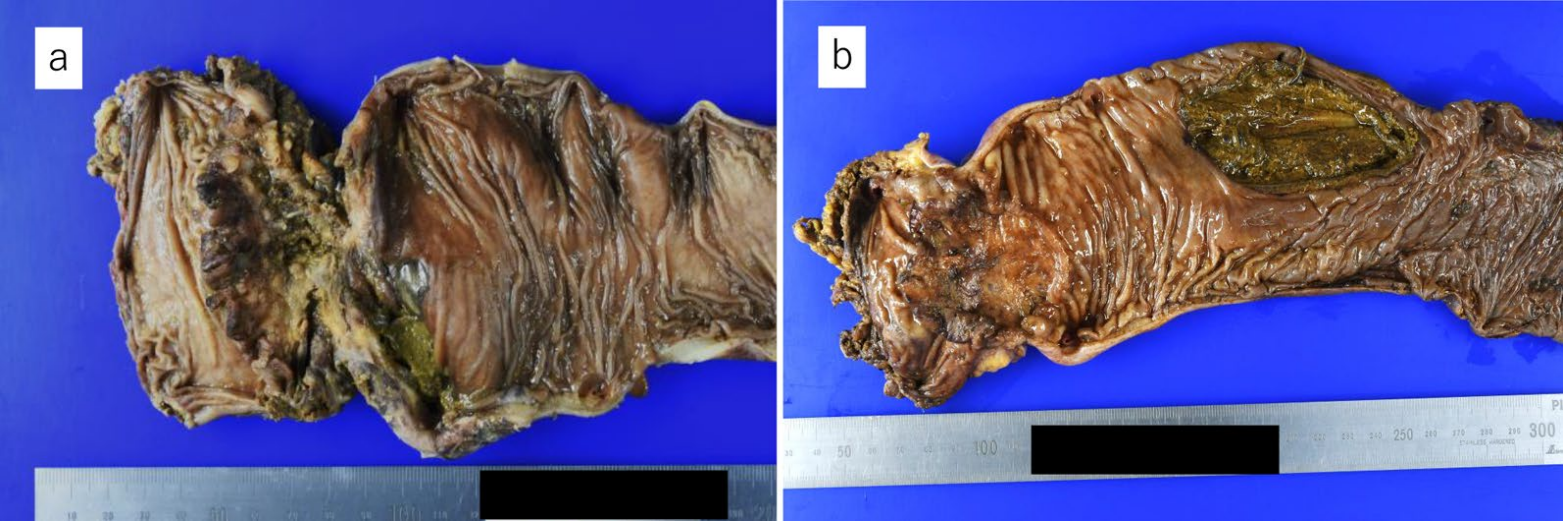
Resected specimen. **a** Case 1: pathological findings were 90 × 56 mm, well-differentiated adenocarcinoma (tub1), pT3, pN2 [5/16] and distal margin of 28 mm. **b** Case 2: pathological findings were 65 × 58 mm, well-differentiated adenocarcinoma (tub1), pT3, pN0 [0/13] and distal margin of 20 mm. A perforation was observed in the rectal sigmoid colon away from the tumor

Therefore, a two-team approach with TaTME was adopted. The details of this technique have been described in other reports [5–7]. Then, en bloc resection of the rectum was completed by collaboration with the transanal team while preserving the autonomic nerves. Finally, the specimen was removed, and the anal side of the rectum was closed with a purse-string suture by the transanal team (Fig. 2). The operative time was 205 min with a small amount of blood loss. On the 7th postoperative day, the drain discharge became cloudy, suggesting the presence of a pelvic abscess. Contrast examination through the drain showed no residual rectum, and the stump rupture was negative. Drainage gradually decreased, the drain was removed on Day 21, and the patient was discharged on Day 25. Pathological examination confirmed tubular adenocarcinoma of the rectum (pT3N2a [5/16]), with negative margins and a complete mesorectum (Fig. 3a). Chemotherapy aimed at conversion surgery for liver metastases was started. As a result, the patient underwent complete liver metastasis resection by “liver resection by associating liver partition and portal vein ligation for staged hepatectomy (ALPPS)” 7 months after the initial surgery. Ten months have passed since the initial surgery, and the patient is still alive without recurrence.

**Fig. 4:**
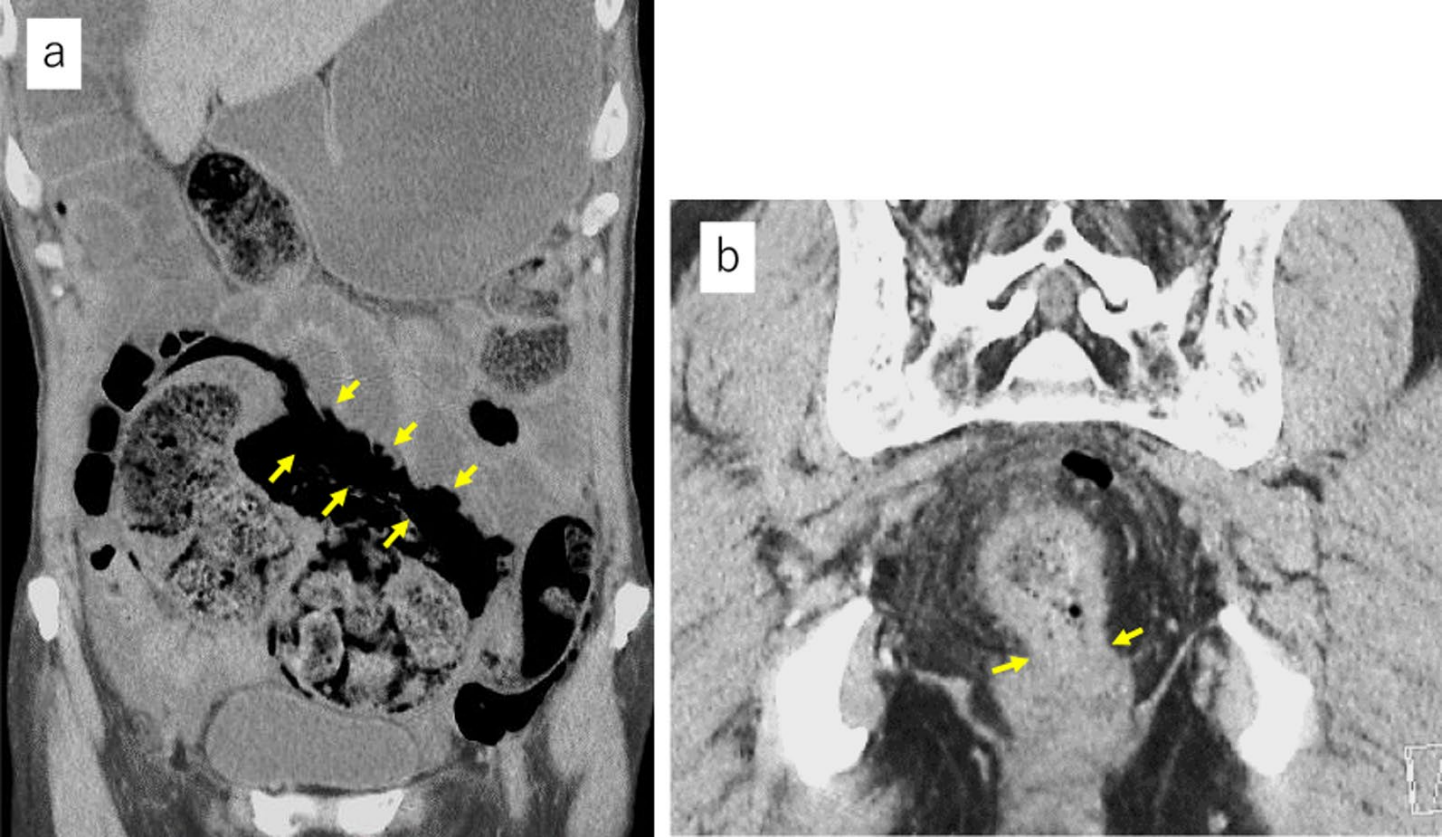
Computed tomography in case 2. **a** The gastrointestinal tract was dilated, and there was a large fecal mass inside the sigmoid colon with free air (yellow arrows) around it. **b** There was wall thickening in the rectum (yellow arrows), and the oral side of the rectum was dilated and had a fecal mass

**Fig. 5:**
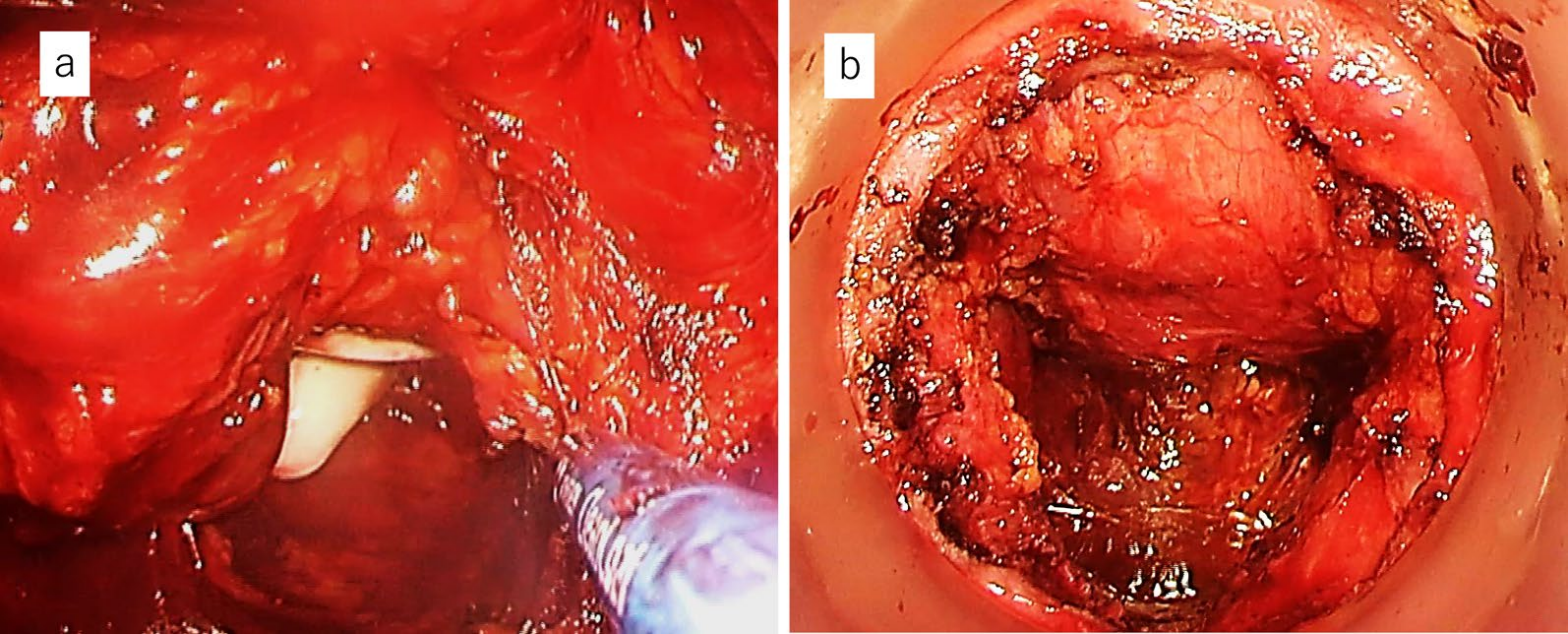
View from the perineal team. **a** The abdominal team assisted the transanal team in performing the surgery in the deep pelvic space. **b** After removal of the specimen, a large prostate was observed from the perineal side through the edge of the anal side of the rectum

*Case 2*: A 75-year-old male who underwent drug therapy for an enlarged prostate experienced abdominal pain after taking laxatives for colonoscopy. After a thorough examination, the patient was diagnosed with obstructive rectal cancer 7 cm from the anal verge and perforation of the rectum on the oral side of the tumor (Fig. 4). He was in a state of septic shock. Emergency open Hartmann’s procedure was performed. The rectum was perforated toward the mesentery, and furthermore, the peritoneum was perforated, and contaminated ascites was found in the peritoneal cavity. He had a narrow pelvis with a bulky tumor, and intestinal dilatation was observed due to fecal impaction. It was believed that dissection of the mesorectum to the anal side of the tumor and transection of the rectum on the anal side of the tumor with a linear stapler would be difficult. Therefore, a two-team approach with TaTME was adopted (Fig. 5). After that, the same procedure was performed as for Case 1. The operative time was 194 min with a small amount of blood loss. Pathological examination confirmed tubular adenocarcinoma of the rectum (pT3N0 [0/10]), with negative margins and a complete mesorectum (Fig. 3b). The postoperative clinical course was good, and the patient was discharged from the hospital on the 17th postoperative day. One year has passed since the surgery, and the patient is currently recurrence-free.

## Discussion

The mortality rate for perforated colorectal cancer is as high as 11–44% [1–3]. Perforated colorectal cancer accounts for 14–21% of colorectal perforations, and such cases have been reported to have a higher mortality rate than other types of perforations [8]. Perforated colorectal cancer also often causes septic shock. Sepsis and frequent postoperative complications exacerbate the condition and increase mortality**.** Even if patients survive the emergency situation, the risk of local recurrence and the survival rate are poor [9, 10].

The operation for perforated colorectal cancer is difficult because of paralytic bowel dilatation with generalized fecal peritonitis, the presence of a bulky tumor, and fecal retention due to obstruction. In addition to performing surgery for life-saving purposes, it is also necessary to aim to cure the cancer. The perforated section should be resected to prevent the patient from being exposed to fecal contents. Resection is necessary to prevent tumor cells from spilling into the abdominal cavity. The 2017 World Journal of Emergency Surgery (WSES) guidelines recommend performing Hartmann’s procedure for perforated colorectal cancer patients in an unstable condition, such as patients with any signs of sepsis or septic shock [11]. The two cases we experienced were in a state of septic shock; thus, we performed Hartmann's procedure. If the perforation is in the rectum, a bulky tumor has to be removed in a narrow pelvis (especially in obese men). Large uterine fibroids and enlarged prostates may worsen the situation. In this study, the first case was a patient with a large uterine fibroid, and the tumor had invaded the peritoneal reflection; the second case was a male patient with a narrow pelvis and a large prostate.

TaTME was first reported by Sylla in 2010 [12] and is gradually being performed worldwide, especially in Europe. This is a novel technique developed to alleviate the difficulty of resecting mid and low rectal cancer, where the pelvis is anatomically strongly inclined. It can help provide a good surgical field even under difficult conditions, and the procedure can be performed linearly from the perineal side [5]. Ensuring a circumferential resection margin (CRM) is essential to decrease the rate of local recurrence [13]. However, ensuring a CRM with TME performed via the traditional transabdominal approach is technically difficult and is oncologically problematic for obese patients and for patients with bulky tumors, a large uterine fibroid, or a large prostate [14, 15]. In such cases, the CRM may be positive. CRM positivity is the factor most associated with local recurrence, and it influences the distant metastasis rate and the overall survival rate [13].

The International TaTME Registry group showed that a CRM is better secured with TaTME than with laparoscopic TME [4]. According to a systematic review that compared the pathological results of TaTME and laparoscopic TME for rectal cancer, TaTME has a lower rate of CRM positivity and is able to secure a larger CRM as well as the distal margin (DM). TaTME is superior in securing the CRM and DM [16]. Moreover, the two-team approach enables the operation to be performed in a coordinated manner from both the abdominal cavity and the perineal side.

Since there are no coherent reports of perforated rectal cancer, the results of the abdominal approach alone are unknown. However, despite the difficulty of the surgery, emergency TaTME allowed us to perform the surgery while securing both the CRM and the DM. There are two limitations to this technique. One is the high manpower requirement. It is difficult to gather enough surgeons to perform a surgery with a two-team approach in an emergency situation. In the two reported cases, we were only able to use the two-team approach from midway into the surgery. Applying the two-team approach from the beginning would have resulted in a significant reduction in the operation time, as well as an overall better outcome and postoperative course for the two patients with septic shock. The other limitation is the lack of long-term oncological results. Case 1 is 10 months post-operation, and Case 2 is 1 year post-operation, so long-term oncological results are not available to show in this case report. Case 1 was treated with chemotherapy for liver metastasis and conversion surgery using ALPPS. Both cases had good short-term results with no recurrence at the time of this report.

## Conclusions

This report presents two cases of perforated rectal cancer treated by emergency TaTME. Although these cases were emergency operations in a situation where it was difficult to pursue radical resection and TaTME may end with only stoma creation, the specimens were safely resected without involvement of the resection margin. Emergency TaTME is considered to be a useful procedure for treatment of perforated rectal cancer.

## Data Availability

Not applicable.

## Abbreviations

TME: Total mesorectal excision
TaTME: Transanal total mesorectal excision
CRM: Circumferential resection margin
DM: Distal margin
